# Synergistic bentonite and *Trichosporon mycotoxinivorans* detoxifier restores humoral immunity to avian influenza vaccination and prevents histopathological lesions in mycotoxin-exposed broiler

**DOI:** 10.14202/vetworld.2026.1796-1810

**Published:** 2026-04-30

**Authors:** Erma Safitri, Tita Damayanti Lestari, Suzanita Utama, Sri Pantja Madyawati, Boedi Setiawan, Rochma Kurnijasanti, Djoko Legowo, Rifdah Aurora, Daryn Arizjinan Febrianti, Anti Febri Hidayani, Siti Darodjah, Goo Jang, Mitsuhiro Takagi

**Affiliations:** 1 Department of Veterinary Science, Division of Veterinary Reproduction, Faculty of Veterinary Medicine, Universitas Airlangga, Surabaya 60115, Indonesia; 2 Department of Veterinary Science, Division of Veterinary Clinic, Faculty of Veterinary Medicine, Universitas Airlangga, Surabaya 60115, Indonesia; 3 Department of Veterinary Science, Division of Veterinary Basic Medicine, Faculty of Veterinary Medicine, Universitas Airlangga, Surabaya 60115, Indonesia; 4 Undergraduate Student of the Faculty Veterinary Medicine, Universitas Airlangga, Surabaya 60115, Indonesia; 5 Department of Veterinary Science, Division of Pathology, Faculty of Veterinary Medicine, Universitas Airlangga, Surabaya 60115, Indonesia; 6 Department of Animal Production, Faculty of Animal Husbandry, Universitas Padjadjaran, West Java, Indonesia; 7 Department of Theriogenology, College of Veterinary Medicine, Seoul National University, Seoul 151-742, Republic of Korea; 8 Laboratory of Theriogenology, Joint Faculty of Veterinary Medicine, Yamaguchi University, Yamaguchi 753-8515, Japan

**Keywords:** aflatoxin B1, avian influenza, bentonite, broiler chickens, humoral immunity, mycotoxin-detoxifier, ochratoxin-A, *Trichosporon mycotoxinivorans*

## Abstract

**Background and Aim:**

Mycotoxin contamination in poultry feed, particularly aflatoxin B1 (AFB1) and ochratoxin-A (OTA), induces immunosuppression and compromises vaccine efficacy, leading to substantial economic losses in broiler production. Avian influenza (AI) remains endemic in several poultry-producing regions, where vaccination is the primary control strategy. However, impaired immune responses due to mycotoxin exposure frequently result in vaccination failure. This study aimed to evaluate the efficacy of a combined mycotoxin-detoxifier containing bentonite and *Trichosporon mycotoxinivorans* in restoring humoral immune responses to AI vaccination and mitigating histopathological lesions in broilers exposed to AFB1 and OTA.

**Materials and Methods:**

A total of 40 broiler chickens were randomly assigned to four groups: negative control (C−), positive control (C+), treatment 1 (T1; 1.1 g/kg detoxifier), and treatment 2 (T2; 1.6 g/kg detoxifier). Groups C+, T1, and T2 received feed contaminated with AFB1 and OTA (0.1 mg/kg each). Birds were vaccinated against AI at 7 days with a booster at 21 days. Antibody titers were measured by hemagglutination inhibition at 14, 21, 28, and 35 days. Histopathological evaluations of the proventriculus and bursa of Fabricius were conducted on day 35. Parametric and non-parametric statistical analyses were applied with significance set at p < 0.05.

**Results:**

Antibody titers were significantly reduced in the mycotoxin-exposed group (C+) compared to the negative control (C−) at all post-vaccination time points. Supplementation with the detoxifier significantly improved antibody titers, particularly in T2, with values comparable to those of the negative control. Histopathological analysis revealed severe inflammatory cell infiltration, oxyntico-peptic cell necrosis, and degeneration in C+, whereas T1 and T2 groups demonstrated significant amelioration of lesions, with T2 showing the greatest protective effect. Similarly, lymphoid follicle depletion and necrosis in the bursa of Fabricius were markedly reduced in detoxifier-treated groups.

**Conclusion:**

Dietary supplementation with a mycotoxin-detoxifier, particularly at 1.6 g/kg, effectively mitigates the immunosuppressive effects of AFB1 and OTA, enhances humoral responses to AI vaccination, and reduces histopathological damage in broilers. This strategy represents a practical and effective approach to improving poultry health and vaccine performance under mycotoxin-contaminated conditions.

## INTRODUCTION

Mycotoxin contamination of poultry feed is a persistent challenge within the global poultry industry. These toxic secondary metabolites, produced by various fungal species, are chemically stable and resistant to degradation, leading to widespread contamination of feed ingredients [1, 2]. Among the numerous mycotoxins of economic and toxicological significance, aflatoxins and ochratoxins are particularly prevalent and pernicious [3, 4]. Accordingly, strategies to reduce mycotoxin exposure are a key important issue, with the inclusion of mycotoxin-detoxifying agents in feed representing a widely adopted and effective approach [5]. A critical consequence of mycotoxin exposure is profound immunosuppression, which severely compromises the efficacy of the vaccination program. Avian influenza (AI) remains an endemic disease of significant concern in numerous poultry-producing countries, including Indonesia, since its emergence in 2003. While vaccination is a cornerstone of AI prevention and control, its success is frequently undermined by factors that impair the host’s immune response. Mycotoxins exert a dose- and duration-dependent immunosuppressive effect, increasing susceptibility to infectious diseases and leading to vaccination failures, even at low levels of chronic exposure. This phenomenon imposes a substantial economic burden on farmers and threatens poultry health.

Animals are also vaccinated as a disease prevention measure, especially against the most common disease affecting broiler chickens, AI. Since its first case report in 2003, AI has been considered endemic in China, Bangladesh, Northeast India, Indonesia, Vietnam, and Egypt [6]. AI clade 2.3.2 has been considered endemic since 2012. Infection with clade 2.3.2 in waterfowl rarely causes clinical symptoms, but in landfowl it can cause severe clinical symptoms [7]. The immunosuppressive effect of mycotoxins impairs the antibody response that should be induced by AI vaccination. The consequences of consuming feed contaminated with mycotoxins include increased susceptibility to infectious diseases, reactivation of chronic infections, and decreased vaccine efficacy [8]. The problem of failed vaccination has been frequently reported by farmers. Exposure to mycotoxins can result in immunosuppressive effects depending on the dose and duration of exposure [9]. Immunity will be affected by continuous exposure to mycotoxins, even when levels remain relatively low. The bursa of Fabricius is a primary lymphoid organ that plays an important role in the maturation and differentiation of B lymphocytes [10]. B lymphocyte cells, which produce antibodies, are an important component of the immune system. Mycotoxins are absorbed in the gastrointestinal tract and then biotransformed in the liver. These biotransformed metabolites are unstable and rapidly bind to the guanine base in DNA. They also interfere with DNA-dependent RNA polymerase activity and reduce RNA and protein synthesis. The biotransformed metabolites of mycotoxins are then haematogenously distributed to organs, including the bursa of Fabricius [11]. The inhibition of synthesis can prevent immune cell proliferation/differentiation, disrupt the production of monokines and interleukins, and consequently impair communication among immune system components [12]. By 2023, the average consumption of chicken meat is expected to reach 6,251 kg/capita/year. The high consumption rate in the community, when compared to beef consumption, can be attributed to the more affordable price. Broiler meat consumption is expected to continue increasing until 2024, reaching 6,407 kg/capita/year [13]. The increase in chicken consumption in Indonesia also affects the population and production of broiler meat as a source of animal protein. Broiler farmers are expected to continue improving maintenance management to produce quality broilers, so that the demand for chicken meat is still met. Mycotoxin contamination in feed leaves residues in the bodies of broilers, especially in the digestive tract [14]. The digestive tract is the first organ exposed to mycotoxins from feed, with aflatoxin B1 (AFB1) potentially having a greater impact than other organs. Intestinal health issues are very common in high-performing poultry lines due to high feed intake, which puts pressure on the digestive system [15]. Chickens have a proventriculus (glandular stomach), which initiates the digestion of feed by secreting hydrochloric acid and digestive enzymes together with a gizzard. Proventriculus forms mucus as a physical barrier to provide protection against pathogens [16]. The glandular stomach is composed of oxyntic and pepsinogen cells, both acid and pepsinogen [17]. Mycotoxins can harm the quality of broiler products and cause financial losses for farmers. Mycotoxin-binding substances can prevent their absorption. This occurs because the binding to mycotoxins prevents their passage from the gut into the bloodstream. Examples of absorbent compounds include cholesterol, complicated indigestible polysaccharides, aluminosilicates, and activated carbon. The use of mycotoxin binders offers an alternative physical method for the breakdown of aflatoxin [18].

However, mycotoxins that have contaminated animal feed cannot be completely removed [19]. One of the most practical approaches is the use of mycotoxin detoxifiers as feed additives. Mycotoxin detoxifiers are divided into two categories: mycotoxin binders, which act as adsorbing agents, and mycotoxin modifiers, which function as biotransformation agents. Toxin binders are widely used due to their safety, ease of application, and cost-effectiveness [20]. Materials that play a role in detoxifying mycotoxins are divided into two types: organic (yeast cell walls, activated charcoal, cholestyramine) and inorganic (hydrated sodium-calcium aluminosilicate, zeolite, bentonite) [21].

Despite extensive research on mycotoxin contamination and mitigation strategies in poultry production, several critical gaps remain insufficiently addressed. Most existing studies have primarily focused on the individual effects of AFB1 or ochratoxin-A (OTA), whereas in practical field conditions, poultry are frequently exposed to multiple mycotoxins simultaneously. The combined toxicological effects of AFB1 and OTA may exhibit additive or synergistic immunosuppressive impacts, which are not adequately represented in studies evaluating single-mycotoxin exposure.

Furthermore, conventional mitigation approaches have largely relied on mycotoxin binders that operate via adsorption. While these agents can reduce toxin bioavailability, they are often limited in their ability to detoxify structurally diverse mycotoxins such as OTA. The emerging use of microbial biotransformation agents, including *Trichosporon* *mycotoxinivorans*, offers an alternative strategy; however, there is limited experimental evidence evaluating the synergistic efficacy of combining adsorption (e.g., bentonite) and biotransformation approaches under controlled *in vivo* conditions.

Another major limitation in current literature is the lack of integrated evaluation of immunological and histopathological outcomes. Previous studies have predominantly assessed performance indicators or biochemical parameters, with relatively few investigations linking mycotoxin exposure to functional immune responses following vaccination, particularly against AI. The impact of mycotoxins on humoral immunity, as measured by antibody titers, remains underexplored in the context of combined detoxification strategies. In addition, there is insufficient information on the correlation between immunosuppression and histopathological alterations in key organs such as the proventriculus and bursa of Fabricius, which play essential roles in digestion and immune cell development, respectively.

Moreover, there is a scarcity of studies evaluating dose-dependent responses of combined detoxifiers under standardized experimental conditions. The absence of such data limits the ability to determine optimal inclusion levels for practical application in poultry feed. Therefore, a comprehensive approach integrating immunological, histopathological, and dose-response assessments under co-exposure to AFB1 and OTA is required to address these knowledge gaps.

This study aimed to comprehensively evaluate the efficacy of a combined mycotoxin-detoxifier containing bentonite as an adsorbing agent and *T. mycotoxinivorans* as a biotransformation agent in broiler chickens exposed to AFB1 and OTA. Specifically, the study was designed to:

1. Assess the impact of combined mycotoxin exposure on humoral immune response by measuring antibody titers following vaccination using the hemagglutination inhibition test.
2. Determine the detoxifier’s ability to restore immune competence by comparing antibody responses between treated and untreated groups during a mycotoxin challenge.
3. Evaluate the protective effects of the detoxifier on the structural integrity of the digestive and immune organs, particularly the proventriculus and bursa of Fabricius, through detailed histopathological examination.
4. Analyze the dose-dependent efficacy of the detoxifier across different inclusion levels to identify the optimal concentration to mitigate mycotoxin-induced immunosuppression and tissue damage.
5. Provide an integrated assessment of immunological and histopathological outcomes to establish the practical relevance of combined detoxification strategies for improving vaccine efficacy and poultry health under field-relevant mycotoxin exposure conditions.

## MATERIALS AND METHODS

### Ethical approval

All experimental procedures involving animals were conducted in accordance with the guidelines for the care and use of laboratory animals and complied with institutional and national regulations on animal welfare. The study protocol was reviewed and approved by the Animal Care and Use Committee of the Faculty of Veterinary Medicine, Universitas Airlangga, Indonesia (Approval No. 1.KEH.033.02.2023).

Broiler chickens (*Gallus domesticus*) used in this study were managed under standard husbandry conditions, with continuous access to feed and water, appropriate environmental control (temperature and lighting), and routine health monitoring throughout the experimental period. All efforts were made to minimize animal stress and discomfort during handling, sampling, and experimental procedures.

Administration of mycotoxin-contaminated feed and detoxifier treatments was carried out under controlled experimental conditions to avoid unnecessary suffering. Blood sampling was performed by trained personnel using aseptic techniques via the brachial vein to minimize pain and tissue injury. The volume and frequency of blood collection were within acceptable limits for poultry.

At the end of the experiment, euthanasia was performed humanely using the cervical dislocation method, as approved by the institutional ethics committee, ensuring rapid loss of consciousness and minimal distress. Tissue collection for histopathological examination was conducted immediately after euthanasia to maintain sample integrity.

No endangered or protected species were involved in this study. The research adhered to the principles of the 3Rs (Replacement, Reduction, and Refinement) by using the minimum number of animals required to achieve statistically valid results and by applying refined experimental techniques to reduce animal suffering.

### Study period and location

The study was conducted from August to December 2024 at the Faculty of Veterinary Medicine, Universitas Airlangga, Surabaya, and at the Molecular Biology Laboratory, Faculty of Mathematics and Natural Sciences, Universitas Brawijaya, Malang, Indonesia.

### Feed preparation with mycotoxin contamination

Mycotoxin exposure was induced through artificially contaminated feed using high-purity laboratory-grade AFB1 standards (product code A-1100, Fermentek Ltd., Jerusalem, Israel) and OTA (product code GC40762, Glpbio, Montclair, CA, USA). Each toxin was added at a concentration of 0.1 mg/kg feed. The compounds were dissolved in ethanol to ensure uniform dispersion and then thoroughly mixed into the base feed using a mechanical mixer. The feed was air-dried to evaporate residual solvent and stored in sealed, moisture-free containers until use.

### Treatment of experimental animals

The research design for this experimental study employed a completely randomized design with four treatment groups and 10 broiler chickens (*G. domesticus*) per treatment group. Mycotoxins and toxin binders were administered through feed for 28 days. Before treatment, broiler chickens were adapted for 7 days and given commercial feed; they were then divided into four treatment groups, with 10 repetitions each.

Broilers were given an AI inactivated vaccine (Medivac® AI H5N1 clade 2.3, PT. Medion Farma, Bandung, Indonesia) by subcutaneous injection at the base of the neck at 7 days of age (0.2 mL/chicken) and an AI booster vaccine at 21 days of age (0.5 mL/chicken) by intramuscular injection. Chicken blood samples were collected at 14, 21, 28, and 35 days of age and subjected to the HI test to assess antibody titers. The HI test used AI antigen clade 2.3.2. HI titers were expressed as log_2_ of the reciprocal of the highest dilution causing complete inhibition.

The improvement in the digestive organ (proventriculus) of broiler samples was evaluated on the 35th day by histopathological examination using hematoxylin–eosin (HE) staining to assess inflammatory cell infiltration, oxyntico-peptic cell necrosis, and oxyntico-peptic cell degeneration.

### Treatment groups

A total of 40 chickens were divided into four treatment groups with ten replications: Negative control group (C−) was the broiler with standard feed; positive control group (C+) was the broiler with standard feed exposed to AFB1 (Fermentek Ltd.) 0.1 mg/kg and OTA (Glpbio) 0.1 mg/kg; treatment groups (T1) and (T2) were broilers given standard feed exposed to AFB1 (Fermentek Ltd.) 0.1 mg/kg and OTA (Glpbio) 0.1 mg/kg contamination and supplemented with mycotoxin-detoxifier agents (Mycofix®, Cat. No. 20026289-FTS, PT. Biomin Indonesia, Jakarta, Indonesia) as feed additives with doses 1.1 g/kg and 1.6 g/kg feed, respectively.

The mycotoxin-detoxifier (Mycofix® Plus 3.0) contains bentonite, diatomaceous earth, Biomin® BBSH® 797, inactivated yeast, phycophytic substances, and plant extract.

### Hen cage and broiler preparation

The cage, room, and other equipment were disinfected using disinfectant one week before the arrival of day-old chicks (DOC). Chicks were housed in battery cages in a controlled environment (initially at 32°C–34°C, gradually reduced to 24°C; 23 h light/day; ad libitum access to feed and water). The basal diet was a commercial starter formulation (Hiprovite CP511, PT. Charoen Pokphand Indonesia, Jakarta, Indonesia) that tested negative for AFB1 or OTA. A total of 40 DOC Cobb strains were reared for 35 days in accordance with broiler rearing standards. During rearing, the chickens were fed a standard starter-stage broiler formulation and given drinking water ad libitum. Before treatment, broiler chickens were adapted for 7 days and given commercial starter feed in the morning and evening, with water ad libitum. Broilers were randomly assigned to battery cages according to treatment groups. Battery cages were maintained until the broilers were 35 days old.

### AI vaccination

Broilers were given an AI inactivated vaccine (Medivac® AI H5N1, PT. Medion Farma) by subcutaneous injection at the base of the neck at 7 days of age (0.2 mL/chicken) and an AI booster vaccine at 21 days of age (0.5 mL/chicken) using the Medivac® ND-AI combination vaccine (PT. Medion Farma) by intramuscular injection.

### Blood serum samples

Chicken blood samples were taken four times at 14, 21, 28, and 35 days of age and subjected to the HI test for antibody titer examination. A total of 1 mL blood samples without anticoagulant were collected through the brachial vein in 40 broiler chickens aged 14, 21, 28, and 35 days. Blood was allowed to clot to obtain serum and transferred into an Eppendorf tube (Sigma-Aldrich, Stl Louis, Missouri, USA).

### Erythrocyte suspension

Erythrocyte suspension was collected from AI antibody-negative donor chickens. Blood was drawn from the brachial vein and collected in EDTA tubes (BD Vacutainer®, Becton Dickinson, Franklin Lakes, NJ, USA). The blood was washed three times using PBS by centrifugation at 1,500 × *g* for 10 min. The buffy coat and plasma were discarded after each wash. The erythrocyte residue was then diluted in PBS to 0.5%.

### Hemagglutination and hemagglutination inhibition tests

The hemagglutination assay was performed to determine the antigen titer to be used in the HI test. The test began by filling microplate wells (A1–A12) with 25 μL of PBS. Then, 25 μL of antigen was added to wells A1 and A12 as antigen controls. The antigen and PBS in well A1 were homogenized using a suction-blow technique with a 25 μL micropipette, and 25 μL of the mixture was transferred sequentially from one well to the next until reaching well A11. The remaining liquid was discarded. All wells (A1–A12) were then filled with 50 μL of 0.5% chicken erythrocyte suspension. The microplate was shaken and incubated at room temperature for 30 min before evaluation.

For duplicate titration, microplate wells numbered 1–5 in rows A and B were filled with 25 μL of PBS. The first well in rows A and B was filled with 25 μL of antigen. The mixture was homogenized and serially diluted up to the fourth well. All wells were then filled with 50 μL of 0.5% erythrocyte suspension, shaken, incubated at room temperature for 30 min, and evaluated.

For serum testing, wells were filled with 25 μL PBS. The first and twelfth wells were filled with 25 μL serum. Serial dilution was performed up to the tenth well. Wells 1–10 were then filled with 25 μL antigen and incubated for 30 min at room temperature. After incubation, 50 μL of a 0.5% erythrocyte suspension was added, and the mixture was incubated for another 30 min before interpretation.

### Proventriculus and bursa of Fabricius sample collection

Sampling was carried out on the 35th day. Chickens were euthanized using the cervical dislocation method. The abdominal cavity was dissected to collect portions of the proventriculus and bursa of Fabricius. Samples measuring approximately 2 × 2 cm were placed in 10% neutral buffered formalin for histopathological preparation using H&E staining.

### Histopathological preparation

The proventriculus and bursa of Fabricius tissues were dehydrated using graded alcohol (70% to absolute), cleared, embedded, sectioned using a microtome, stained using HE, and mounted.

### Histopathological observations

Histopathological evaluation was performed by a blinded pathologist. Five non-overlapping fields per slide were scored at 400× magnification. Microscopic examination assessed inflammatory cell infiltration, oxyntico-peptic cell necrosis, and degeneration (Table 1). The scoring system was as follows: 0 = no change; 1 = mild (0–10%); 2 = moderate (10–50%); and 3 = severe (>50%) per field of view [22, 23]. The scoring criteria for lymphoid follicle depletion and necrosis in the bursa of Fabricius are presented in Tables 2 and 3 [24].

**Table 1:** Proventriculus damage scoring parameters [22, 23].

Lesion shape	Score	Description
Inflammatory cell infiltration	0	No change
	1	Mild, >0–10% in one field of view
	2	Moderate, 10–50% in one field of view
	3	Severe, >50% in one field of view
Oxyntico-peptic cell necrosis	0	No change
	1	Mild, >0–10% in one field of view
	2	Moderate, 10–50% in one field of view
	3	Severe, >50% in one field of view
Oxyntico-peptic cell degeneration	0	No change
	1	Mild, >0–10% in one field of view
	2	Moderate, 10–50% in one field of view
	3	Severe, >50% in one field of view

**Table 2:** Scoring criteria for lymphoid follicle depletion in the bursa of Fabricius [24].

Score	Identification of lymphoid follicle depletion
0	No lymphoid follicle depletion observed
1	Mild, 1–30% depletion per field of view
2	Moderate, 31–50% depletion per field of view
3	Severe, ≥51% depletion per field of view

**Table 3:** Scoring criteria for lymphoid follicle necrosis in the bursa of Fabricius [24].

Score	Identification of lymphoid follicle necrosis
0	No necrosis observed
1	Mild, 1–30% necrosis per field of view
2	Moderate, 31–50% necrosis per field of view
3	Severe, ≥51% necrosis per field of view

### Statistical analysis

The HI test data were organized and assessed for normality using the Shapiro–Wilk test. Data conforming to a normal distribution were analyzed using a one-way analysis of variance followed by Duncan’s multiple range post hoc test. Histopathological scores were analyzed using the Kruskal–Wallis test, with pairwise comparisons conducted using the Mann–Whitney U test. All statistical analyses were performed using SPSS version 31.0.0.0 for Windows (IBM Corp., Armonk, NY, USA), with significance determined at p < 0.05 [25].

## RESULTS

### AI antibody titer in broiler chickens exposed to mycotoxin: AFB1 and OTA

A positive HI test was indicated by the absence of hemagglutination, characterized by the sedimentation of erythrocytes upon tilting the microplate. Conversely, the presence of hemagglutination was indicated by the lack of erythrocyte sedimentation at the bottom of the microplate well. The maximum serum dilution that prevented hemagglutination against the antigens was also used to calculate the HI titer.

**Figure 1:**
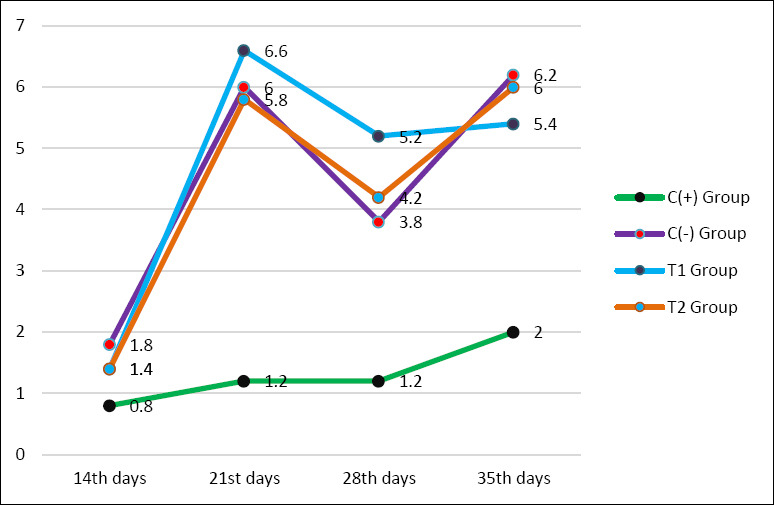
Average antibody titers of each group aged 14, 21, 28, and 35 days are shown in the chart. The C+ group is the positive control group indicated by the green line with black markers, C− group is the negative control indicated by the purple line with red markers, T1 group is fed with exposure to aflatoxin B1 0.1 mg/kg + ochratoxin 0.1 mg/kg + mycotoxin binder 1.1 g/kg shown by the blue line with purple markers, and T2 group is fed with exposure to aflatoxin B1 0.1 mg/kg + ochratoxin 0.1 mg/kg + mycotoxin binder 1.6 g/kg shown by the orange line with blue markers.

The HI test results showed that the use of a mycotoxin-detoxifier in feed exposed to mycotoxins affected the antibody titer of broiler chickens. Antibody titers in chickens aged 21, 28, and 35 days differed significantly, whereas at 14 days or 1 week after vaccination, antibodies to AI had not yet formed. There was a significant difference (p < 0.05) between the C− group at the 2nd (6.00 ± 1.22^b^), 3rd (3.80 ± 0.83^b^), and 4th (6.20 ± 3.27^b^) weeks post-vaccination and the C+ group at the 2nd (1.20 ± 0.83^a^), 3rd (1.20 ± 0.83^a^), and 4th (2.00 ± 0.707^a^) weeks post-vaccination, but the C− group was not significantly different (p > 0.05) compared with T1 and T2 groups at the 2nd, 3rd, and 4th weeks post-vaccination (Table 4 and Figure 1).

**Table 4:** Average antibody titer (log₂) of broiler chicken post-vaccination.

Group	1 Week (14^th^ day)	2 Weeks (21st day)	3 Weeks (28^th^ day)	4 Weeks (35^th^ day)
C+ Positive Control	0.80 ± 0.83^a^	1.20 ± 0.83^a^	1.20 ± 0.83^a^	2.00 ± 0.707^a^
C− Negative Control	1.80 ± 1.30^a^	6.00 ± 1.22^b^	3.80 ± 0.83^b^	6.20 ± 3.27^b^
T1 (Treatment 1)	1.40 ± 0.54^a^	6.60 ± 0.89^b^	5.20 ± 2.38^b^	5.40 ± 2.30^b^
T2 (Treatment 2)	1.40 ± 0.89^a^	5.80 ± 0.44^b^	4.20 ± 0.83^b^	6.00 ± 1.22^b^

Different superscripts indicate significant differences between treatment groups (p < 0.05). C− = commercial feed, C+ = feed with exposure to aflatoxin B1 0.1 mg/kg + ochratoxin 0.1 mg/kg, T1 = feed with exposure to aflatoxin B1 0.1 mg/kg + ochratoxin 0.1 mg/kg + mycotoxin binder 1.1 g/kg, and T2 = feed with exposure to aflatoxin B1 0.1 mg/kg + ochratoxin 0.1 mg/kg + mycotoxin binder 1.6 g/kg.

Following a transient decrease at 28 days, antibody titers increased again by day 35. The T2 group (6.00 ± 1.22^b^) demonstrated a significantly higher antibody titer compared to the C+ group (2.00 ± 0.707^a^). Although the highest reported values (T2) are below or close to the established protection threshold of 7 log_2_, the titer values were not significantly different from those of the negative control (C−). Changes in the antibody titer of broiler chickens over time are shown in Table 4 and Figure 1.

### Improvement of proventriculus

**Figure 2:**
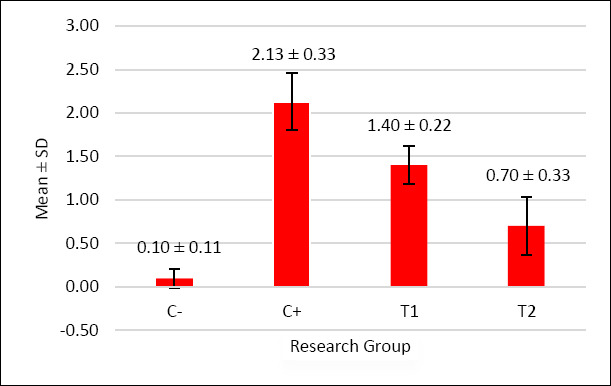
The graph illustrates the mean score of oxyntico-peptic cell necrosis in the proventriculus of broiler chickens. The negative control group C− exhibited almost no necrosis, while the positive control group C+ fed with AFB1- and OTA-contaminated feed showed the highest necrosis score. The administration of mycotoxin-detoxifier in the treatment groups T1 (1.1 g/kg) and T2 (1.6 g/kg) significantly reduced necrosis compared to C+. Among them, T2 showed the lowest level of necrosis, approaching that of the negative control C−.

**Figure 3:**
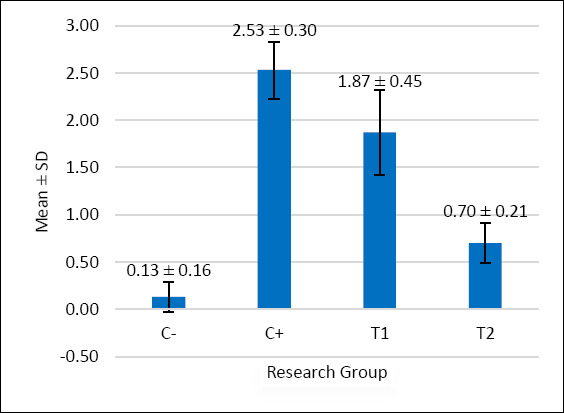
The graph illustrates the mean score of inflammatory cell infiltration in the proventriculus of broiler chickens. The negative control group C− showed minimal infiltration. In contrast, the positive control group C+ that received AFB1- and OTA-contaminated feed demonstrated the highest infiltration score. The treatment groups, T1 (1.1 g/kg) and T2 (1.6 g/kg), exhibited significantly lower infiltration levels compared to C+. Among them, T2 showed the most reduced infiltration, approaching the condition observed in the negative control group.

**Figure 4:**
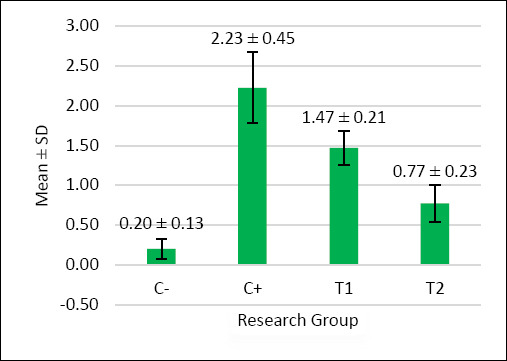
The graph illustrates the mean score of oxyntico-peptic cell degeneration in the proventriculus of broiler chickens. The negative control group C− showed very low degeneration, indicating normal cellular conditions. The positive control group C+, exposed to AFB1 and OTA, displayed the highest degeneration scores, signifying severe cellular damage. Treatment groups T1 (1.1 g/kg) and T2 (1.6 g/kg) exhibited significantly lower degeneration compared to C+. Among these, T2 showed the least degeneration and was closer to the condition of the negative control group.

**Figure 5:**
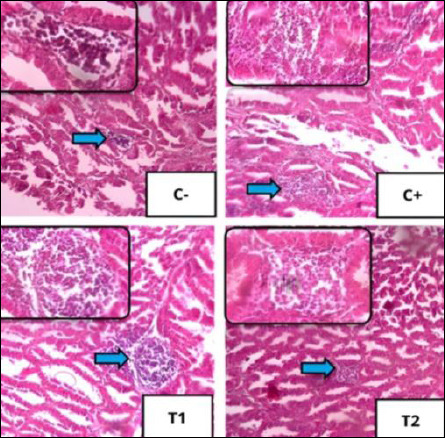
Histopathological features of inflammatory cell infiltration using 100× magnification, with 400× magnification insert using hematoxylin–eosin staining. Infiltration of polymorphonuclear/mononuclear inflammatory cells in each treatment in the proventriculus of broiler chickens is shown by blue arrows.

**Figure 6:**
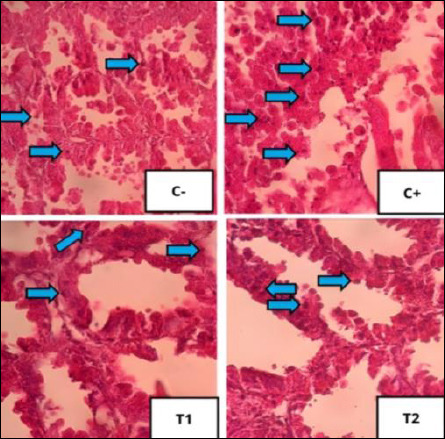
Histopathological features of oxyntico-peptic cell necrosis using 400× magnification with hematoxylin–eosin staining. Oxyntico-peptic cells that experience necrosis show that the cell nucleus undergoes three changes, namely pyknosis (the cell nucleus becomes dark, dense, and small), karyorrhexis (the cell nucleus is divided into several segments), or karyolysis (the cell nucleus fades because it has completely lysed), as indicated by the blue arrow.

**Figure 7:**
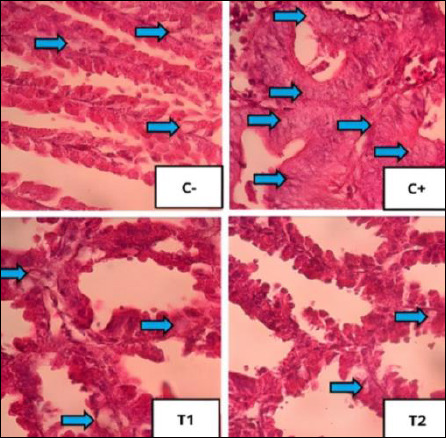
Histopathological features of oxyntico-peptic cell degeneration using 400× magnification with hematoxylin–eosin staining. Blue arrows indicate degeneration of oxyntico-peptic cells of the proventriculus. Oxyntico-peptic cells that experience degeneration appear cloudy, enlarged, and thecytoplasm is filled with water vacuoles.

The scoring results of inflammatory cell infiltration, oxyntico-peptic cell necrosis, and oxyntico-peptic cell degeneration of the proventriculus of broiler chickens are presented in Table 5, and the graphs are illustrated in Figures 2–4, and then the histopathological features are illustrated in Figures 5–7.

**Table 5:** Scoring results for proventricular inflammatory cell infiltration, oxyntico-peptic cell necrosis, and degeneration in broiler chickens exposed to mixed mycotoxin and mycotoxin binder.

Group	Inflammatory cell infiltration (Mean ± SD)	Oxyntico-peptic cell necrosis	Oxyntico-peptic cell degeneration
C−	0.13 ± 0.16^a^	0.10 ± 0.11^a^	0.20 ± 0.13^a^
C+	2.53 ± 0.30^d^	2.13 ± 0.33^d^	2.23 ± 0.45^d^
T1	1.87 ± 0.45^c^	1.40 ± 0.22^c^	1.47 ± 0.21^c^
T2	0.70 ± 0.21^b^	0.70 ± 0.33^b^	0.77 ± 0.23^b^

Different superscripts in the same column indicate significant differences between treatments (p < 0.05).

Based on Table 5, the inflammatory cell infiltration scores showed significant differences (p < 0.05) among treatment groups: C− (0.13 ± 0.16^a^), C+ (2.53 ± 0.30^d^), T1 (1.87 ± 0.45^c^), and T2 (0.70 ± 0.21^b^). The results of the analysis showed that administration of mycotoxin binder was most effective in T2, with values close to the C− group.

Based on Table 5, the oxyntico-peptic cell necrosis score showed significant differences (p < 0.05) among treatment groups: C− (0.10 ± 0.11^a^), C+ (2.13 ± 0.33^d^), T1 (1.40 ± 0.22^c^), and T2 (0.70 ± 0.33^b^). The results showed that T2 was the most effective dose.

Based on Table 5, the oxyntico-peptic cell degeneration scores showed significant differences (p < 0.05) among treatment groups: C− (0.20 ± 0.13^a^), C+ (2.23 ± 0.45^d^), T1 (1.47 ± 0.21^c^), and T2 (0.77 ± 0.23^b^). The T2 group again showed the best improvement.

### Improvement of bursa of Fabricius

**Figure 8:**
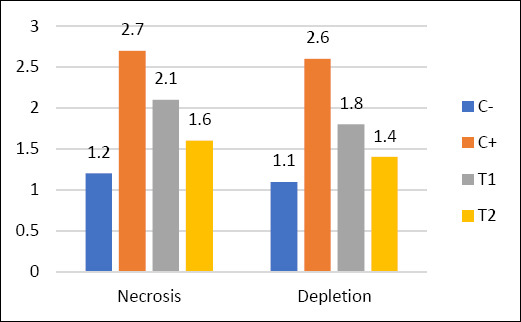
Mean values of lymphoid follicle necrosis and depletion in different treatment groups (C− = negative control, C+ = positive control, T1 and T2 = treatment groups).

**Figure 9:**
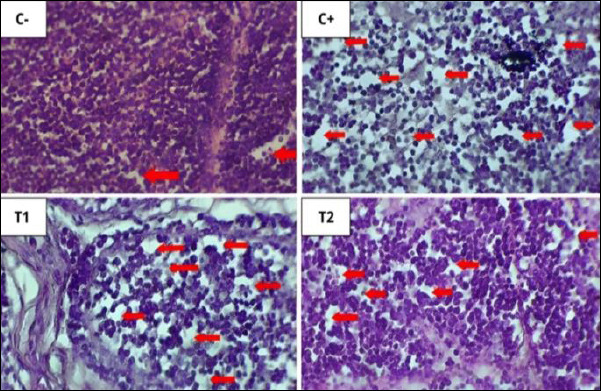
Histopathological features of lymphoid follicular depletion in the bursa of Fabricius under 400× magnification with HE staining. The red arrow indicates lymphoid follicle depletion.

**Figure 10:**
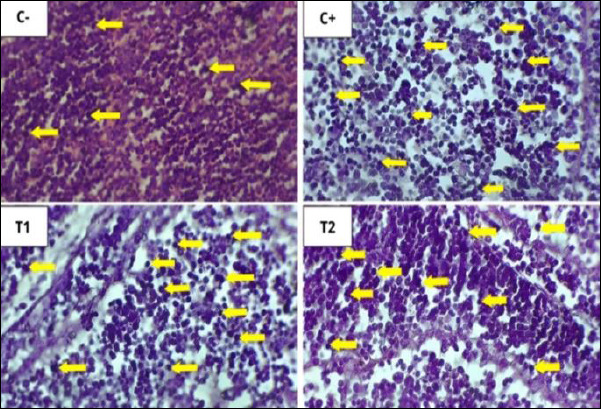
Histopathological features of lymphoid follicular cell necrosis in the bursa of Fabricius under 400× magnification with HE staining. The yellow arrow indicates lymphoid follicle cell necrosis. The results of statistical analysis using the Kruskal–Wallis test showed a significant difference between treatment groups (p < 0.05). In the Mann–Whitney test, the C− group showed the lowest depletion and necrosis compared to other treatment groups, whereas the C+ group showed the highest depletion and necrosis.

The mean and standard deviation of lymphoid follicle depletion and necrosis are shown in Table 6, and the graphs are illustrated in Figure 8, with histopathological features shown in Figures 9 and 10.

**Table 6:** Mean and standard deviation of lymphoid follicle depletion and necrosis.

Group	Necrosis (Mean ± SD)	Depletion (Mean ± SD)
C−	1.2 ± 0.16^a^	1.1 ± 0.10^a^
C+	2.7 ± 0.24^d^	2.6 ± 0.41^d^
T1	2.1 ± 0.48^c^	1.8 ± 0.19^c^
T2	1.6 ± 0.27^b^	1.4 ± 0.30^b^

Different superscripts in the same column indicate significant differences (p < 0.05).

## DISCUSION

### Epidemiological context and study rationale

Since around 2012, Indonesia has faced endemic H5N1 AI clade 2.3.2. This virus continues to circulate in commercial, semi-commercial, and free-range poultry farms, resulting in consistently high infection pressure. At the same time, local feed is often contaminated with multiple mycotoxins (e.g., aflatoxin, deoxynivalenol, fumonisin), which can cause immunosuppression and reduce the vaccination response. Due to this combination, many farmers report vaccination failures despite routine vaccination programs. Therefore, this research was conducted to address these challenges.

### Antibody response following vaccination

The treatment group with the highest antibody titer at 1 week after vaccination was the negative control group, with a titer of 1.80. This value has not reached 7 log_2_ as the protective AI antibody titer [26]. The minimum AI antibody titer in poultry in the field is 7 log_2_ to protect against mortality, and 7 log_2_ is also considered a protective titer to reduce virus replication and transmission, whereas a titer ≤ 4 log_2_ can only protect chickens by 40% against the virus. The positive control group, which was only fed contaminated feed, had the lowest mean titer of 0.80.

The antibody titer in broiler chickens at 2 weeks after vaccination increased, as AI antibody titers typically peak 2 weeks after vaccination [27]. The antibody titer in chickens at 3 weeks post-vaccination was lower than at 2 weeks post-vaccination, indicating that the antibody titer decreased after booster administration. The decrease was still close to the protective AI antibody titer in the C−, T1, and T2 treatment groups. The average antibody titer at 35 days of age (4 weeks post-vaccination) was higher than at 28 days of age (3 weeks post-vaccination). The C− group achieved the highest antibody titer of 6.20 ± 3.27^b^, which was not significantly different from T2 (6.00 ± 1.22^b^). In the 1st week after vaccination, the body is still at the stage of recognizing the incoming antigen. The low average antibody titer across the four treatment groups is due to the newly formed immune response, so it takes longer to reach a protective titer [28].

### Effect of mycotoxins on immune suppression

In poultry, OTA decreases the size of the primary lymphoid organs, namely the thymus and bursa of Fabricius, which produce T and B cells, respectively [29]. Broiler chickens fed diets containing 0.1 mg OTA/kg for 35 days showed decreased thymus weight and reduced serum protein fractions [30]. The low antibody levels may be due to OTA inhibition of protein production [31]. OTA production is influenced by carbon and nitrogen sources, and it inhibits protein synthesis through its effects on phenylalanine t-RNA synthase and phenylalanine hydroxylase [32].

The mechanism of immunosuppression by AFB1 is similar to that of OTA, namely through inhibition of protein synthesis. Aflatoxins are converted *in vivo* into active metabolites that bind to DNA and RNA, impair DNA-dependent RNA polymerase activity, and inhibit RNA and protein synthesis. This inhibition disrupts lymphocyte proliferation, cytokine production, and immunoglobulin synthesis [33].

### Mechanism of mycotoxin-detoxifier action

The addition of mycotoxin-detoxifier as a feed additive is one of the most common ways to reduce mycotoxin exposure. Mycotoxin detoxifiers function as binders (adsorption) and modifiers (biotransformation). Binders reduce toxin bioavailability in the gastrointestinal tract, whereas modifiers convert toxins into less toxic compounds [34]. Bentonite acts as a binder for AFB1, while *T. mycotoxinivorans* functions as a biotransformation agent for OTA. The polar interaction between the toxin and the binder facilitates toxin excretion [35]. OTA, being a nonpolar molecule, requires biotransformation strategies using microorganisms to degrade it into less-toxic metabolites [36, 37].

### Vaccine response dynamics and influencing factors

The slow increase in antibody titers is associated with oil-based adjuvants in inactivated vaccines, which act as antigen depots and delay antigen release [28]. Adjuvants enhance immune responses, improve antigen presentation, and support immune responses in susceptible populations [38]. Variability in antibody response can be influenced by antigen quality, vaccine strain differences, and adjuvant composition [39].

### Antibody kinetics and study limitations

The decrease in antibody titers at 28 days may be related to antibody half-life [40]. Antibody half-life reflects the time required for antibody levels to decline to half of peak values, and titers alone do not fully indicate protection. This observation may also relate to study limitations, including a small sample size (40 birds), the absence of challenge testing, a short study duration, the evaluation of a single mycotoxin combination, and a lack of performance parameters. Additionally, the absence of confirmation of actual mycotoxin concentrations in feed and the lack of analytical validation (e.g., HPLC, enzyme-linked immunosorbent assay) represent limitations.

### Inflammatory response in proventriculus

Inflammation is a natural defense mechanism involving multiple cellular processes to eliminate harmful stimuli [41]. Inflammatory cells include polymorphonuclear (PMN) and mononuclear (MN) cells [42]. Acute inflammation involves neutrophils and macrophages, whereas chronic inflammation involves lymphocytes and fibroblasts [43]. The observed infiltration may result from immune responses, environmental stressors, or the presence of foreign material [42, 43].

### Necrosis of oxyntico-peptic cells

Necrosis refers to irreversible cell death resulting from pathological injury, often due to ischemia [44]. Nuclear changes include pyknosis, karyorrhexis, and karyolysis [42]. Acute aflatoxicosis is known to induce necrosis [45].

### Degeneration of oxyntico-peptic cells

Degeneration is a reversible form of cellular injury that may progress to necrosis if the damage persists [43]. Degenerated cells appear swollen with vacuolated cytoplasm [46].

### Histopathological changes in bursa of Fabricius

Administration of OTA and AFB1 caused lymphoid follicle necrosis and depletion in the bursa of Fabricius [47]. Necrosis exceeds regeneration, leading to depletion and structural damage [48]. Lymphoid disintegration begins in the medulla and progresses to the cortex, resulting in follicular atrophy [49].

### Effect of detoxifier on bursal protection

Groups T1 and T2 showed reduced depletion and necrosis compared with C+ and C−. The dose of 1.6 g/kg was most effective in reducing histopathological damage. Detoxifiers reduce toxin absorption and systemic distribution, protecting immune organs such as the bursa of Fabricius [50]. Mycotoxin binders form complexes with toxins and facilitate their excretion, reducing bioavailability *in vivo* [51].

### Role of detoxifier components

Bentonite and diatomaceous earth exhibit strong adsorptive capacity, reducing toxin absorption [52]. *T. mycotoxinivorans* converts OTA into non-toxic metabolites [53, 54]. Microbial detoxification pathways include deacetylation, oxidation, and glucosylation [55–57].

### Dose-dependent detoxification efficacy

Detoxifier supplementation at 1.1 g/kg and 1.6 g/kg significantly reduced necrosis and depletion. The higher dose showed greater efficacy in limiting toxin absorption and systemic distribution.

## CONCLUSION

This study demonstrated that exposure to AFB1 and OTA significantly impairs humoral immune responses to AI vaccination and induces marked histopathological damage in the proventriculus and bursa of Fabricius of broiler chickens. The positive control group consistently exhibited the lowest antibody titers and the most severe tissue alterations, confirming the immunosuppressive and cytotoxic effects of mycotoxins. In contrast, supplementation with mycotoxin-detoxifier improved immune responses and reduced tissue damage, with the higher dose (1.6 g/kg) showing the most pronounced effect. Antibody titers in the T2 group approached those of the negative control and were significantly higher than those of the positive control, while histopathological lesions, including inflammatory infiltration, necrosis, and degeneration, were substantially reduced.

From a practical perspective, the findings highlight the importance of incorporating mycotoxin detoxifiers into poultry feed, particularly in regions where multi-mycotoxin contamination is common. The combined use of adsorption (bentonite) and biotransformation (*T. mycotoxinivorans*) mechanisms provides a more comprehensive strategy to mitigate the adverse effects of mycotoxins, thereby enhancing vaccine efficacy and improving poultry health and productivity. This approach can help reduce economic losses associated with vaccination failure and disease susceptibility in commercial poultry production systems.

A major strength of this study is the integrated evaluation of immunological (antibody titer) and histopathological parameters under controlled *in vivo* conditions, providing a comprehensive understanding of both functional and structural impacts of mycotoxin exposure and detoxification. Additionally, the use of dose-based treatment groups allowed identification of an effective inclusion level of the detoxifier.

However, several limitations should be considered. The study was conducted with a relatively small sample size and over a short duration, and included no viral challenge trial to directly assess protective efficacy. The investigation was limited to a single combination and concentration of mycotoxins, and performance parameters such as growth rate and feed efficiency were not evaluated. Furthermore, the absence of analytical confirmation of mycotoxin concentrations in feed and the lack of detailed analytical methods (e.g., HPLC or ELISA) may limit the precision of exposure assessment.

Future studies should include larger sample sizes, longer experimental durations, and challenge trials to validate protective immunity under field conditions. Evaluation of multiple mycotoxin combinations, different detoxifier formulations, and their effects on production performance and economic outcomes would provide broader applicability. In addition, incorporating analytical verification of toxin levels and mechanistic studies at molecular and immunological levels would further strengthen the evidence base.

In conclusion, dietary supplementation with a mycotoxin detoxifier, particularly at 1.6 g/kg feed, effectively mitigates the detrimental effects of AFB1 and OTA on immune function and tissue integrity in broiler chickens. This strategy offers a practical and effective solution to improve vaccine responsiveness and overall poultry health in mycotoxin-contaminated production environments.

## DATA AVAILABILITY

The data generated during the study are included in the manuscript.

## AUTHORS’ CONTRIBUTIONS

ES: Conceptualized and supervised the study and edited and revised the manuscript. TDL, SU, SPM, BS, RK, DL, and SD: Designed and conducted the study and statistical data analysis. RA, DAF, AFH: Conducted the study and data collection and analysis, and drafted the manuscript. GJ and MT: Statistical analysis, visualization, and drafted and revised the manuscript. All authors have read and approved the final version of the manuscript.
